# Supplementary material for: Intervalley scattering by acoustic phonons in two-dimensional MoS2 revealed by double-resonance Raman spectroscopy
Source: Nat Commun. 2017 Mar 9;8:14670. doi: 10.1038/ncomms14670 (PMC5347091; doi:10.1038/ncomms14670)
Supplement: Supplementary Information — Supplementary Figures, Supplementary Tables, Supplementary Notes and Supplementary References [file ncomms14670-s1.pdf]

SUPPLEMENTARY INFORMATION

Supplementary Note 1: Electronic band structure and phonon dispersion

Supplementary Figure 1a shows the phonon dispersion of monolayer (1L) MoS_2 calculated by density functional theory (DFT) within the generalized gradient approximation (GGA-PBE). The horizontal dashed lines at 454 and 460 cm^{-1} indicate the frequencies of p_2 and p_3. The line at 227 cm^{-1} indicates frequency of p_2 halved, which is close to the frequency of the van Hove singularity along $\overline{\mathrm{KM}}$ at 225.2 cm^{-1} (see panel to the right). The van Hove singularity originates from a saddle point in the LA-branch phonon dispersion: the LA phonon state at about midway along $\overline{\mathrm{KM}}$ is a local minimum along $\overline{\mathrm{KM}}$, but is a local maximum along the direction perpendicular to that. The line at 233 cm^{-1} indicates the frequency of p_3 halved, which is close to the frequency of LA(**K**) at 233.7 cm^{-1} (see panel to the right). Besides, the halved frequency of p_4 lies at 235 cm^{-1}, close to the LA(**M**) phonon frequency. The above is consistent with the identification of p_2 as the van Hove singularity and p_3 and p_4 as 2LA($\sim$ **K**) and 2LA($\sim$ M).

Supplementary Figure 1b shows the DFT electronic band structure of monolayer, bilayer and bulk MoS_2. As the number of layer increases, a conduction band state midway along $\overline{\mathbf{K\Gamma}}$, labeled **Q**, drops in energy and aligns with the conduction band minimum at the **K** point. The phonon that connects these two states has a wave vector of **M** (see inset in Supplementary Fig. 1b, left panel). Therefore, the alignment brings the following second-order process into resonance: an electron is scattered from **K** to **Q** and then back by two LA phonons at the **M** point. Thus, this trait allows us to assign the p_4 peak, whose intensity is stronger in bulk MoS_2, to 2LA($\sim$ M).

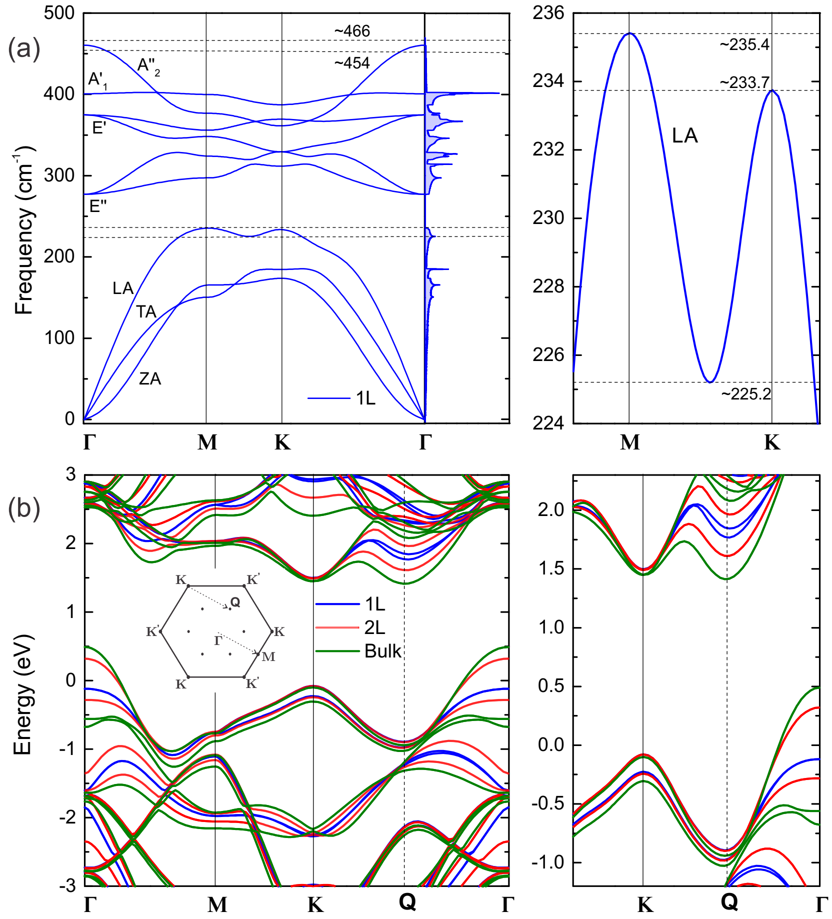

Supplementary Figure 1: **Electronic and phonon dispersion in MoS_2.** (a) Right panel: Phonon dispersion and phonon density of states for 1L-(blue) MoS_2. Left panel: Close-up of the LA-phonon branch along $\overline{\mathrm{MK}}$. The calculated phonon dispersion is converged to within $\sim$1 cm^{-1} in terms of plane-wave energy cutoff, k-point sampling, and supercell size. (b) Right panel: The electronic band structure of 1L-(blue), 2L-(red) and Bulk (green) MoS_2. As the layer number increases, the conduction band state at **Q** lowers until it aligns with the conduction band minimum at the **K** point. A schematic of the first Brillouin zone is shown in the inset with the symmetry points labeled. Left panel: Close-up of the state at **Q** point.

The opposite trend occurs for the electronic band structure of the WS_2. The alignment of the conduction band state at **K** and **Q** already occurs for the monolayer. As the number of layer increases, the state at **Q** drops in energy and the alignment is broken [1].

Supplementary Note 2: Raman spectrum at 1.94 eV for 1L, 2L, 3L and bulk MoS_2

To further confirm our conclusion of p_4 intensity dependence, we compare the Raman spectrum of 1L, 2L, 3L and bulk MoS_2 at 1.94 eV (close to the A exciton energy). Notice that in Supplementary Fig. 2, the shape of the 2LA band for 1L MoS_2 sample is different from the shape of this band for 2L, 3L and bulk. For 1L MoS_2, the shoulder (black arrow) appears in the right side of the band, showing that p_4 is weaker than p_2 and p_3, as expected for a direct gap system. On the other hand, for 2L, 3L and bulk, the shoulders appear in the left side, showing that p_4 is stronger than p_2 and p_3, in agreement with the fact that they are indirect band-gap semiconductors.

As shown in Fig. 1 of the main text, the intensity of p_4 at 2.11 eV spectrum increases for 1L MoS_2, and becomes comparable with p_1 (but still weaker than p_2 and p_3). Note that 2.11 eV is $\sim$0.2 eV above the A exciton energy, which is exactly the energy misalignment between the conduction band state at **Q** and **K** (see Supplementary Fig. 1b). That is, near 2.11 eV, the conduction band state at **Q** becomes a resonant intermediate state (with a potentially large electron-phonon matrix element between **K** and **Q**). This explains the non-negligible intensity of p_4 near 2.11 eV.

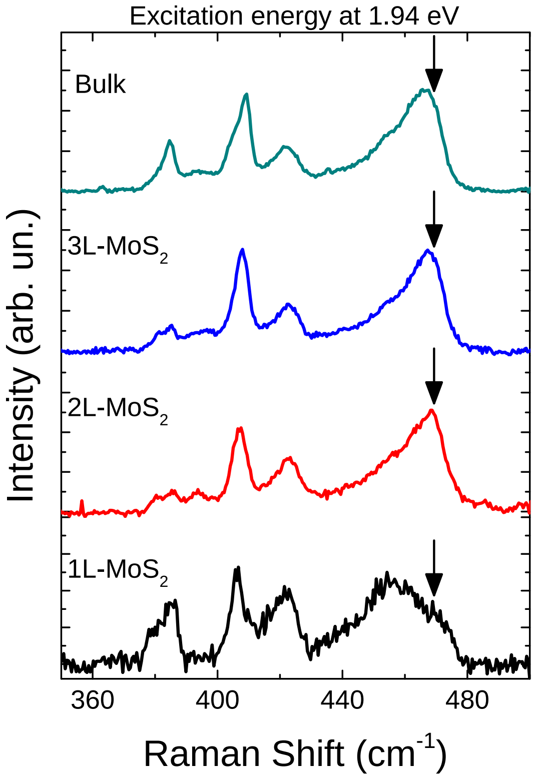

Supplementary Figure 2: **Raman spectrum.** Raman spectrum at 1.94 eV excitation energy for 1L, 2L, 3L and bulk MoS_2. The back arrow indicates the p_4 intensity dependence.

Supplementary Note 3: Dispersion from the B exciton in Bulk MoS_2

As mentioned in the main article, as the laser energy increases past the A exciton towards the B exciton in bulk MoS_2 (from 1.89 to 2.05 eV), the calculated LA($\sim$ **K**)+TA($\sim$ **K**) phonon red-shifts from 424 cm^{-1} to 416 cm^{-1}. As the laser energy further increases past the B exciton in bulk MoS_2 (2.05–2.17 eV), a second branch of dispersion should occur, where LA($\sim$ **K**)+TA($\sim$ **K**) restarts from 424 cm^{-1} and red-shifts towards 416 cm^{-1} again. The observation of this second dispersion from a very weak Raman peak has been reported by Sekine *et al.* [2] at a temperature of

7 K. In Supplementary Fig. 3a and b, we compare the result adapted from Sekine's paper and the calculated dispersion of the LA($\sim$ **K**)+TA($\sim$ **K**) phonon. The energy of the band gap used in the calculation is adjusted to match the optical gap of bulk MoS_2 at 7 K.

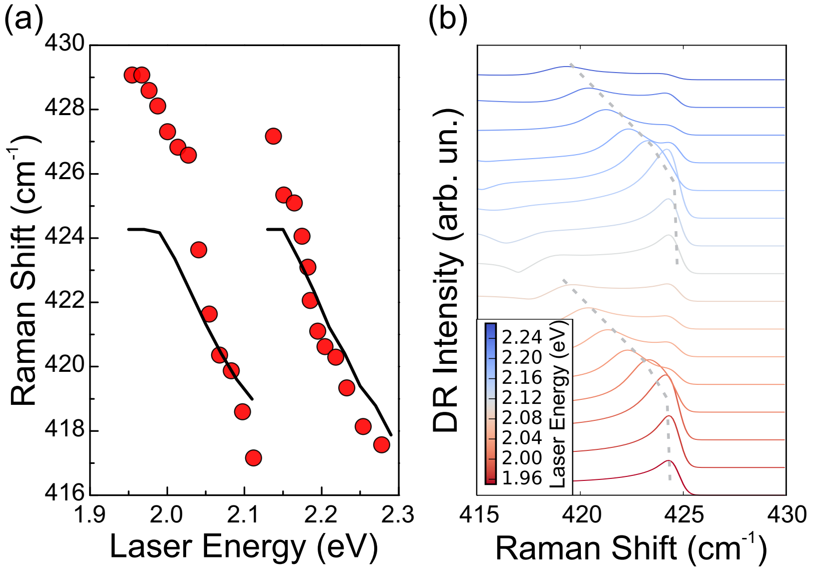

Supplementary Figure 3: **Dispersion assuming B excitons in bulk MoS_2.** (a) The calculated DR intensity and (b) dispersion of bulk MoS_2 including the two top-most valence bands from Ref. 2.

Supplementary Note 4: Comparative Raman spectra of 1L, bulk and, defective 1L MoS_2

Supplementary Fig. 4 shows the Raman spectra of 1L and bulk (for 1.94, 2.04 and 2.11 eV) and the defective monolayer (for 1.92, 2.06 and 2.14 eV) in the spectral range of 200–500 cm^{-1}. As can be seen, the pristine samples (1L and bulk) do not show any defective band around 230 cm^{-1}. Notice that in the defective sample, the 2LA band broadens and decreases in intensity, which is expected since the disruption of the pristine lattice decreases phonon lifetimes and introduces new

defective Raman bands within this range.

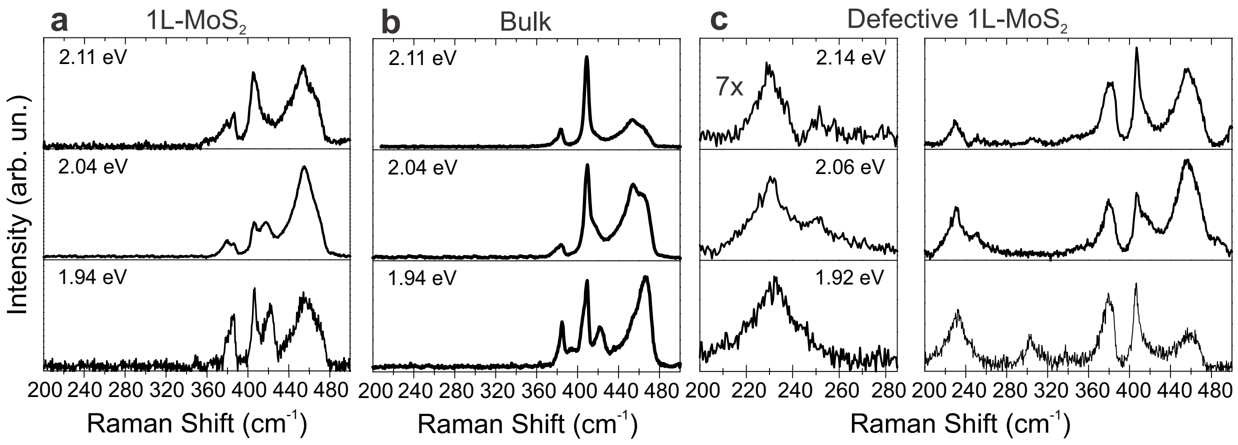

Supplementary Figure 4: **Comparative Raman spectra of 1L, bulk and defective 1L MoS_2.** Raman spectra of (a) 1L and (b) bulk for three excitation energies (1.94, 2.04 and 2.11 eV) and (c) defective 1L MoS_2 also for three excitation energies for (1.92, 2.06 and 2.14 eV). Notice that no defective Raman band appears for the pristine samples (1L and bulk).

Supplementary Note 5: Relationship between single-particle and excitonic models

Although the experimental resonant Raman spectra for both 1L and bulk MoS_2 (Fig. 1c,d in the main text) show that the phonons near 460 cm^{-1} are on-resonance for excitation energies near the A and B excitons, the resonance appears suppressed for 1L MoS_2 near the A exciton, *i*.e. Fig. 1c shows a lack of red contours near 460 cm^{-1} at the A exciton level when compared to the same region for bulk. Apart from the 2LA(**M**) being off-resonance for 1L (see text above), this suppression may also be attributed to the forbidden intervalley scattering of holes at the A exciton level.

The spin-polarized valence bands of monolayer MoS_2 host different channels for hole scattering. In the single-particle picture, as shown in Supplementary Fig. 5e where phonons are represented by horizontal line due to their small energies, holes in the A exciton (left, red) cannot be scattered to the opposite valley (right, blue) due to the required spin-flip. However, holes in the B exciton reside on the second-highest valence band (left, blue) and can be scattered to the top-most valence band of the opposite valley (right, blue) while conserving spin.

The same process applies to the excitonic picture, where the selection rules for scattering electrons or holes in the single-particle picture are carried over by the expansion coefficients of the excitonic wave function (expansion into single-particle states), *i*.e. exciton scattering is forbidden if the hole component of the excitonic wavefunction changes spin. For the example, in Supplementary Figure 5d, A excitons with $\boldsymbol{\mathcal{Q}} = 0$ created at the $\mathbf{K}$ valley, and state $|\mathrm{eh}\rangle = |\mathbf{KK}\rangle$, cannot be scattered to the state $|\mathrm{eh}\rangle = |\mathbf{KK'}\rangle$ by altering the hole component, however B excitons with $\boldsymbol{\mathcal{Q}} = 0$ are allowed to be scattered to the $|\mathbf{KK'}\rangle$ state by altering the hole component.

Considering that the double-resonant Raman intensity for 2LA phonons is dominated by intervalley scattering of valence band holes (see the section on symmetry arguments), the suppression of the second-order resonances at the A exciton energy for monolayer samples can be attributed to hole scattering being forbidden at this energy. This suppression is not observed for multilayer MoS_2 due to availability of both spin components at the A exciton energy. We also note in Supplementary Fig. 5f that the on-resonance phonon disperses monotonically and continuously, which justifies the use of spin-unpolarized electronic structures in our calculation.

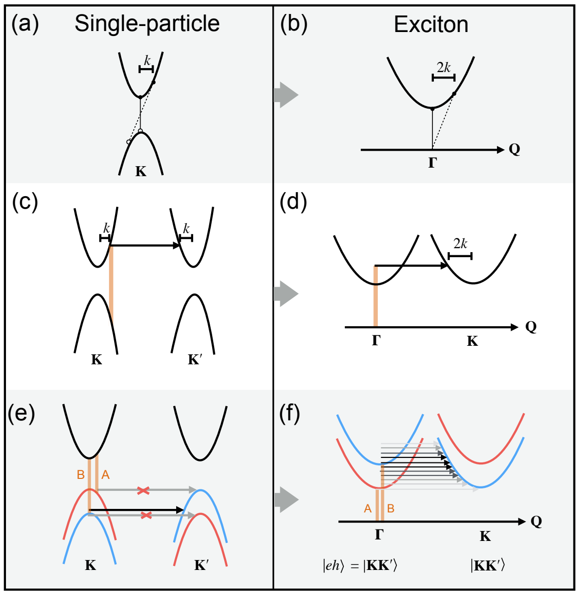

Supplementary Figure 5: **Intervalley scattering as modelled using single-particle and excitonic band structures.** (a) In single-particle bands assuming (for convenience) equal effective masses m^* for electrons and holes, excitons can be represented by pairing electrons and holes with matching group velocities at $\mathbf{k}_\mathrm{e} = -\mathbf{k}_\mathrm{h} \equiv \mathbf{k}$, yielding an exciton with a center-of-mass momentum of $\boldsymbol{Q} = 2\mathbf{k}$. (b) The same excitonic states as shown in (a) are represented here in the exciton band structure whose band curvature is half that of the single-particle case, reflecting the exciton mass being $M = 2m^*$. Intervalley scattering can be represented either (c) between single-particle states or (d) between excitonic states. Given the same excitation energy $E_\mathrm{L} = E_\mathrm{g} + 2k^2/2m^*$, the on-resonance phonons in both systems are away from **K** by $2\mathbf{k}$. (e,f) Same as (c,d), but now considering the two topmost spin-polarized valence bands in 1L MoS_2. Red (blue) indicate spin-up (spin-down) for single-particle states in (e) and for the hole component of the excitonic state in (f). Intervalley hole scattering events are only allowed from the second valence band in one valley to the first valence band in the other valley.

Supplementary Note 6: Symmetry discussion for the proposed assignments

The assignments proposed in the main text, 2LA($\sim$ **K**) and LA($\sim$ **K**)+TA($\sim$ **K**), are further supported by group theory and electron-phonon coupling matrix elements calculated within DFT for electrons and holes exactly at **K**; *i*.e. the following should only apply to the phonon scattering processes at a laser energy of 1.9 eV, where only the electronic states at **K** are on-resonance (hence the symbol "$\sim$" will not be used in this section). 1L MoS_2 has $C_{3\text{h}}$ group symmetry at the **K** point and its electronic states at the valence band maximum and conduction band minimum at $\mathbf{K}(\mathbf{K}')$ has $\mathrm{A}'(\mathrm{A}')$ and $\mathrm{E}_2{}'(\mathrm{E}_1')$ symmetry[3]. The electron-phonon coupling matrix element for an intervalley process at the **K** point, $M_{\text{el-ph}} = \left\langle \mathbf{K}'|\hat{\mathrm{H}}_{\text{e-ph}}(\mathbf{K})|\mathbf{K} \right\rangle = \left\langle \mathbf{K}|\hat{\mathrm{H}}_{\text{e-ph}}(\mathbf{K}')|\mathbf{K}' \right\rangle^*$ can only be non-vanishing if the phonon of the representation E_2' for scattering conduction band electrons and A' for scattering valence band holes. Table 1 shows the phonons representation at the $\boldsymbol{\Gamma}$, **M** and **K** points obtained by applying all symmetry operations to each phonon eigenvector calculated within DFT. The calculated representations satisfy the compatibility relations given in Ref. 4. Therefore the only acoustic phonon that satisfies the selection rule is the LA(**K**), for the scattering valence band holes.

This result is consistent with the calculated electron-phonon coupling matrix elements $M_{\text{el-ph}}$ for acoustic phonons at **K** using wave functions and phonon modes obtained from DFT, where the only non-vanishing matrix element is the scattering of valence band hole via LA(**K**) phonons, as shown in the last two columns in Table 1. In addition, this analysis explains why the double-resonance Raman intensity of the 2LA(**K**) is strong: the intensity of TA(**K**)+LA(**K**) is weaker

because the TA(**K**) phonon does not satisfy the selection rule, but can be still have non-zero scattering amplitudes as a result of the phonons *not exactly* at the **K** point.

Supplementary Table 1: Phonon symmetry at **Γ**, **M** and **K** points in monolayer MoS_2. Electron-phonon coupling matrix elements connecting intervalley valence band and conduction band states are given in eV.

f/ cm^{-1}	**Γ** (D_{3h})	f/ cm^{-1}	**M** (C_{2v})	f/ cm^{-1}	**K** (C_{3h})	$\langle cb\|\hat{H}_{e\text{-}ph}(\mathbf{K})\|cb\rangle$	$\langle vb\|\hat{H}_{e\text{-}ph}(\mathbf{K})\|vb\rangle$
460	A_2''	400	A_1	387	E_1'	0	0
401	A_1'	377	B_2	369	A'	0	0.012
375	E'	356	B_1	361	E_2''	0	0
375		348	A_1	329	A''	0	0
277	E''	324	B_2	329	E_2'	0.061	0
277		297	A_2	312	E_1''	0	0
0		235	A_1	234(LA)	A'	0	0.146
0	$E'+A_2''$	165	B_2	185(TA)	E_1'	0	0
0		151	B_1	174(ZA)	E_2''	0	0

Supplementary Table 2: Character table of the C_{3h} group, [$e = \exp(2\pi\, i/3)$].

	E	$C_3(z)$	C_3^2	σ_h	S_3	$(\sigma_h C_3^2)$
A'	1	1	1	1	1	1
E'_1	1	e	e^*	1	e	e^*
E'_2	1	e^*	e	1	e^*	e
A''	1	1	1	-1	-1	-1
E''_1	1	e	e^*	-1	$-e$	$-e^*$
E''_2	1	e^*	e	-1	$-e^*$	$-e$

Supplementary Table 3: Character table of the C_{2v} group.

	E	$C_2(z)$	$\sigma_v(xz)$	$\sigma_v(yz)$
A_1	1	1	1	1
A_2	1	1	-1	-1
B_1	1	-1	1	-1
B_2	1	-1	-1	1

Supplementary References

1. Berkdemir, A. *et al.* Identification of individual and few layers of WS_2 using Raman spectroscopy. *Sci. Rep.* **3**, 1755 (2013).

2. Sekine, T., Uchinokura, K., Nakashizu, T., Matsuura, E., & Yoshizaki, R. Dispersive Raman mode of layered compound 2H-MoS_2 under the resonant condition. *J. Phys. Soc. Jpn.* **53**, 811–818 (1984).

3. Kormányos, A. *et al.* Trigonal warping, the Γ valley, and spin-orbit coupling effects. *Phys. Rev. B* **88**, 045416 (2013).

4. Ribeiro-Soares, J. *et al.* Group theory analysis of phonons in two-dimensional transition metal dichalcogenides. *Phys. Rev. B* **90**, 115438 (2014).
